# Assessing Eligibility for Anticancer Drug Health Insurance Reimbursement Using Large Language Models: Benchmark Development and Comparative Study

**DOI:** 10.2196/95877

**Published:** 2026-06-15

**Authors:** Junhyuk Seo, Taerim Kim, Ju-Hyun Kim

**Affiliations:** 1 Healthcare Research Institute, ETOILE Inc, Seoul, Republic of Korea; 2 Department of Digital Health, Samsung Advanced Institute of Health Sciences and Technology (SAIHST), Sungkyunkwan University, Seoul, Republic of Korea; 3 Department of Emergency Medicine, Sungkyunkwan University School of Medicine, Samsung Medical Center, Seoul, Republic of Korea; 4 Department of Obstetrics and Gynecology, University of Ulsan College of Medicine, Asan Medical Center, 88, Olympic-ro 43-gil, Songpa-gu, Seoul, 05505, Republic of Korea, 82 10-9668-6227

**Keywords:** large language models, health insurance, reimbursement, eligibility verification, anticancer drugs, clinical decision support, benchmark, natural language processing, gynecologic cancer, National Health Insurance

## Abstract

**Background:**

Administrative costs in the health care system are driven in part by complex insurance eligibility determinations. Large language models (LLMs) are increasingly used for health insurance–related queries, yet their reliability for structured logical reasoning over coverage criteria has not been systematically evaluated.

**Objective:**

This study aimed to develop a benchmark for anticancer drug reimbursement eligibility determination and evaluate whether LLMs can reliably perform eligibility verification.

**Methods:**

We constructed a benchmark based on South Korea’s National Health Insurance reimbursement guidelines for 3 gynecologic cancers (cervical, uterine, and ovarian), using a tristate adjudication framework (eligible, ineligible, and undeterminable). Three gynecologic oncology experts and a utilization review nurse validated the benchmark. Six LLMs from 3 providers (Anthropic, Google, and OpenAI) were evaluated using the official guideline document as input. Each case was evaluated 3 times per model, with final predictions determined by majority vote, and performance was compared across the 3 outcome classes.

**Results:**

The benchmark comprises 74 anticancer regimens with 222 cases. Overall verification accuracy ranged from 77.9% to 88.7% across the 6 models. Eligible and ineligible cases were classified with high recall (86.5%‐98.6%), but undeterminable cases showed a marked decline across all models (44.6%‐70.3%). Performance varied by cancer type, with uterine cancer showing the lowest undeterminable recall (16.7%), corresponding to the highest guideline complexity. Undeterminable cases were predominantly misclassified as eligible rather than ineligible. The tristate framework enabled logic-based error analysis of 235 incorrect predictions, revealing information gap-filling as the dominant failure pattern (n=196, 83.4%), followed by criterion misapplication (n=20, 8.5%) and false uncertainty (n=19, 8.1%). Subtype analysis indicated that information gap-filling errors were concentrated at hierarchical elements of the guideline. Sensitivity analyses showed that converting the guideline document to structured text degraded performance, while web search–enabled condition (0%‐3.2% tool invocation across models) and structure-guided prompting did not produce significant changes from baseline.

**Conclusions:**

In this benchmark, LLMs classified clearly eligible and ineligible cases with relatively high recall but showed limited reliability on undeterminable cases. The dominant error pattern was information gap-filling, in which models inferred eligibility rather than withholding judgment. These findings indicate that LLMs, in their current form, should be deployed as supervised decision-support tools rather than as independent adjudicators in reimbursement review.

## Introduction

In the United States, billing- and insurance-related activities were estimated to cost approximately US $471 billion annually, representing roughly 18% of national health expenditure [1]. At the provider level, billing- and insurance-related costs account for 3% to 25% of professional revenue depending on the type of clinical encounter [2]. This administrative burden poses significant negative impacts for both patients and clinicians [3,4]. Although health systems and payer models vary worldwide, billing processes follow a common pathway—from eligibility verification and coding to claim submission and rework—across countries with diverse payer structures [5]. These burdens persist even in single-payer systems, where centralized rule-based reimbursement introduces a different form of administrative complexity.

In South Korea, the entire population is enrolled in the National Health Insurance (NHI) by law and relies on predefined coverage rules and eligibility criteria [6]. Under this system, the National Health Insurance Service functions as the single insurer, while the Health Insurance Review & Assessment Service (HIRA) conducts centralized claims review and quality assessment [7]. Reimbursement decisions are made through a retrospective claims review process, in which submitted claims are evaluated for compliance with detailed coverage rules [8,9]. Under this retrospective review structure, eligibility determination becomes a high-stakes post hoc decision, where errors can directly lead to claim rejection, payment adjustment, and downstream administrative burden.

As anticancer agents continue to be developed and covered by the NHI, national expenditure on these drugs surged by 168.2% from 2013 to 2022, reaching US $982 million in 2022 [10]. Alongside this rapid cost increase, eligibility criteria for anticancer drugs have become increasingly complicated, requiring simultaneous assessment of multiple clinical variables—such as tumor type, prior regimen sequences, biomarker thresholds, and performance scores—connected by nested AND/OR conditions [11]. This combinatorial structure makes manual review prone to omission and misinterpretation, particularly when multiple interdependent conditions must be evaluated simultaneously.

A recent report found that users of ChatGPT in the United States alone send 1.6 to 1.9 million health insurance–related messages each week to understand coverage and navigate claims and denials [12]. Despite this high level of use, large language models (LLMs) often produce hallucinations and other errors that can lead to critical mistakes, especially in medical domains [13,14]. When LLMs have been applied to improve the understandability of clinical guidelines, unintended omissions and changes in meaning were identified in up to 20% of revised subsections [15]. If even surface-level revisions can introduce errors, the risk may be amplified when LLMs must reason over the logical structure of guidelines to reach coverage determination.

Recent studies have shown that LLMs can support reimbursement-related tasks, such as detecting clinical conditions for appropriate coding [16], and automating *International Classification of Diseases* code assignment from clinical documentation [17]. However, these applications focus on information extraction from clinical notes. Determining reimbursement eligibility, by contrast, requires structured logical reasoning in which multiple clinical attributes must be jointly assessed against complex rule sets, where a single incorrect inference can invalidate the entire decision. Moreover, in clinical practice, available documentation is often incomplete [18,19], and a reliable system must also recognize when the evidence is insufficient to reach a determination rather than forcing a binary decision. Yet, no standardized benchmark exists to evaluate these capabilities under real-world insurance review constraints.

To address this gap, we developed a benchmark for anticancer drug reimbursement eligibility based on South Korea’s national guidelines. Our benchmark formalizes the condition-level adjudication logic that clinicians and utilization review nurses routinely apply when evaluating incomplete clinical evidence against coverage rules. It covers 3 gynecologic cancers—cervical, uterine, and ovarian—and includes eligible, ineligible, and undeterminable cases to assess not only correctness but also whether models can recognize when available evidence is insufficient to support a determination. Using this benchmark, we evaluated LLM reliability for reimbursement eligibility adjudication under incomplete information.

## Methods

### Benchmark Development

**Figure 1.**
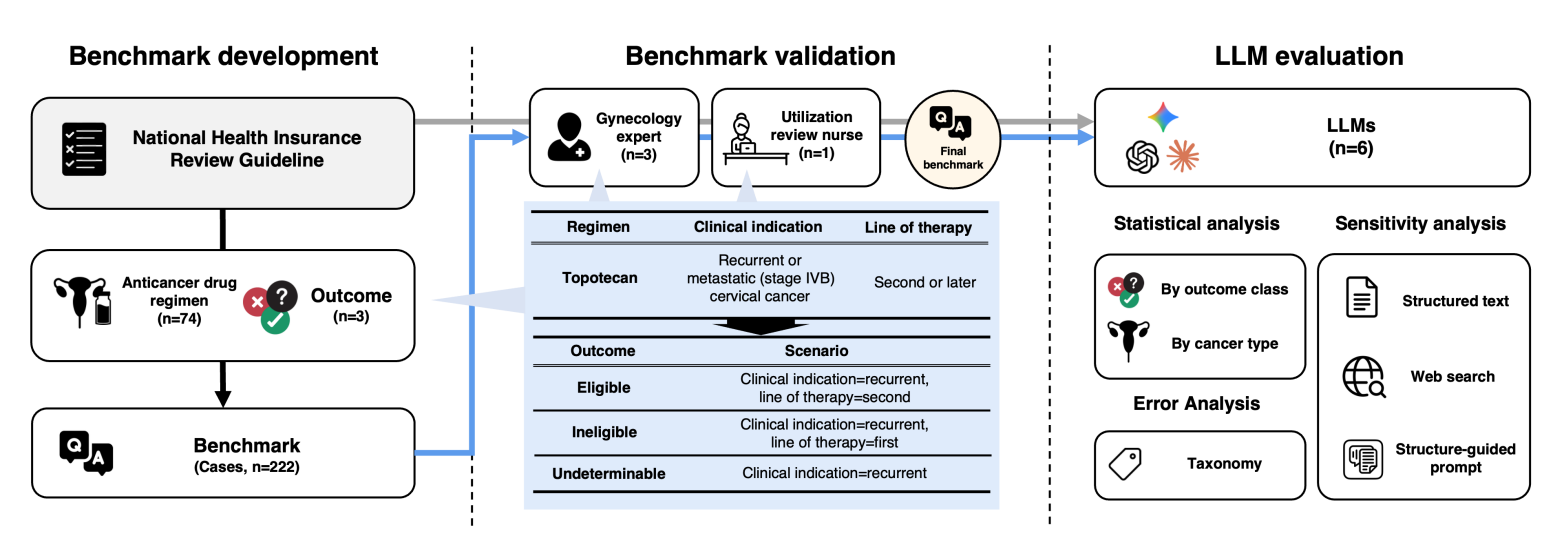
Study design overview. The benchmark comprises 222 cases derived from national reimbursement guidelines for 3 gynecologic cancers (74 regimens × 3 outcome classes). Validation was performed by 3 gynecologic oncology experts and an independent utilization review nurse. Six models were evaluated using the finalized benchmark. LLM: large language model.

The study design is summarized in Figure 1. This study was reported in accordance with the TRIPOD-LLM (Transparent Reporting of a Multivariable Prediction Model for Individual Prognosis or Diagnosis-Large Language Model) [20]. A completed checklist is provided in Checklist 1.

We developed the benchmark using the HIRA reimbursement review guidelines for anticancer drugs, version dated February 1, 2026. Three major gynecologic cancers—cervical cancer, uterine cancer, and ovarian cancer—were included. For each cancer type, reimbursement criteria were translated into structured clinical attributes representing heterogeneous patient scenarios. Candidate benchmark items were created by a researcher with expertise in medical informatics (JS).

**Figure 2.**
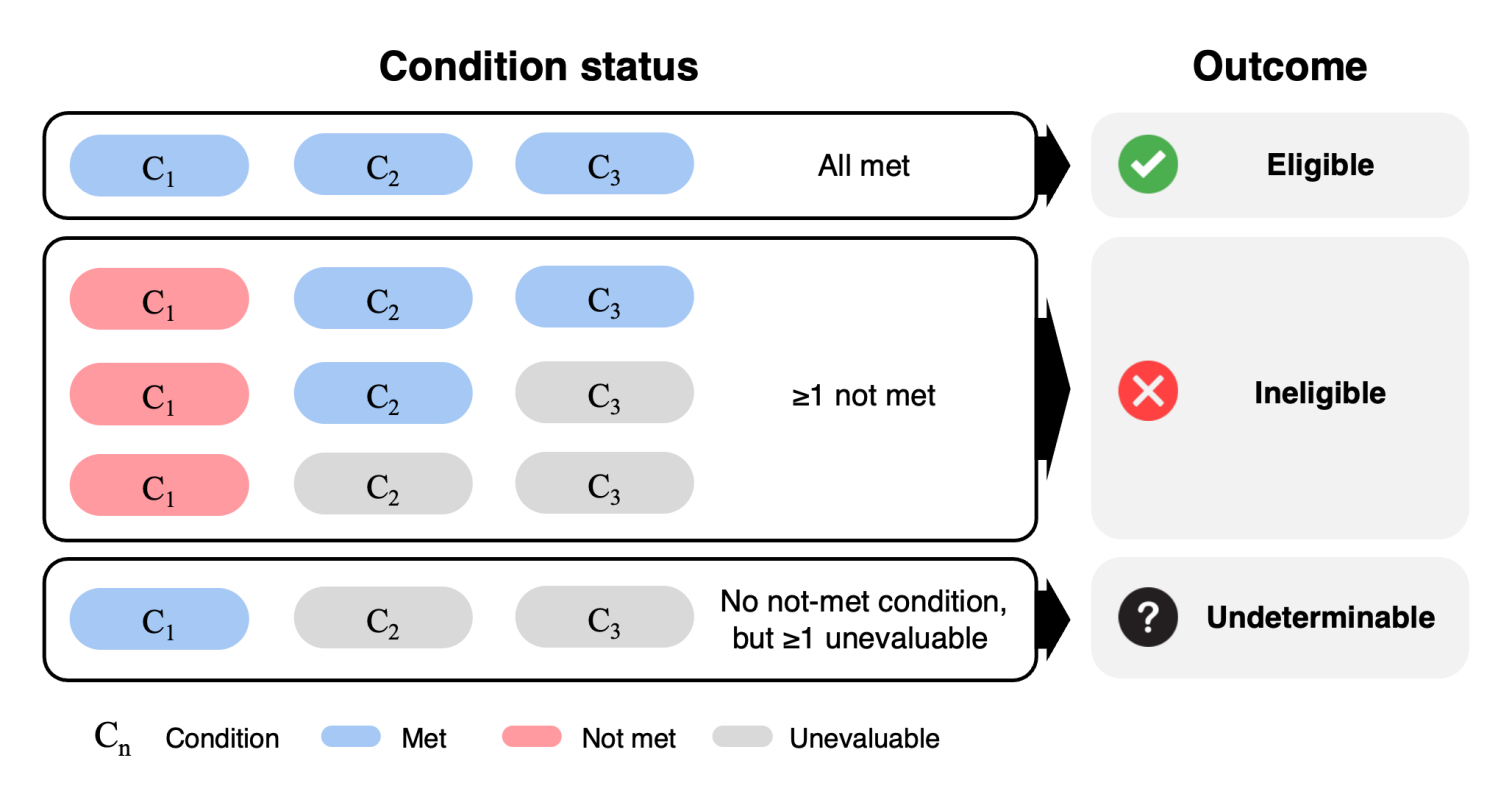
Tristate adjudication framework for benchmark outcome assignment. Each required condition for a regimen was classified as met, not met, or unevaluable. Eligible cases met all required conditions. Ineligible cases included at least 1 explicitly not-met condition. Undeterminable cases included no explicitly not-met condition but at least 1 unevaluable condition due to missing information.

The benchmark was constructed within a tristate adjudication framework, including eligible, ineligible, and undeterminable outcomes for each anticancer regimen. This outcome assignment is based on 3 different condition-level states: met, not met, and unevaluable. We use “not met” to denote a condition whose required value is present but violates the criterion, and “unevaluable” to denote a condition that cannot be assessed because the required information is absent (Figure 2). The unevaluable state was included to reflect real-world documentation contexts in which information required for applying a coverage criterion may be absent, incomplete, or inaccessible.

For instance, a case is classified as ineligible when 1 or more conditions are explicitly not met, regardless of whether other conditions are unevaluable. A case is classified as undeterminable when no condition is explicitly not met, yet 1 or more conditions remain unevaluable. To control task difficulty, ineligible and undeterminable cases were designed as near-miss scenarios containing only 1 to 2 conditions not met or unevaluable. The detailed definitions of the outcomes are shown in Table 1.

**Table 1.** Definitions of eligibility outcomes used in the benchmark, with illustrative examples from cervical cancer topotecan (single use).

Outcome	Definition	Example scenario	Explanation
Eligible	All required conditions are met	Clinical indication=recurrent; line of therapy=second	All guideline conditions are satisfied.
Ineligible	≥1 condition is explicitly not met, regardless of whether other conditions are unevaluable	Clinical indication=recurrent; Line of therapy=first	Line of therapy is first line. The condition is present but violates the criterion (“not met”).
Undeterminable	No condition is explicitly not met, but ≥1 condition is unevaluable due to absent clinical information	Clinical indication=recurrent	Line of therapy is required but absent. No condition is violated, so the case cannot be classified as ineligible, yet a determination cannot be reached (“unevaluable”).

### Benchmark Validation

The benchmark was reviewed by 3 gynecologic oncology experts, including 2 nurse practitioners and 1 physician. Validation focused on the clinical plausibility of the patient scenarios, the appropriateness of attribute combinations, and the consistency of the reference answers.

For instance, the experts identified that palliative regimens inherently target patients with recurrent or metastatic disease, meaning that clinical indication is implicitly satisfied, and its removal alone does not produce a valid undeterminable case. For such regimens, additional clinical attributes had to be removed to construct undeterminable scenarios. The review also identified certain regimens that are permissively reimbursed in clinical practice despite not strictly meeting the guideline criteria. For these cases, reference answers were assigned based on the guideline criteria.

After clinical expert review, all benchmark cases were independently reviewed by a utilization review nurse, and discrepancies were resolved through consensus while preserving the balanced class structure. Interrater agreement between experts and the utilization review nurse was assessed using Cohen κ. This multidisciplinary validation approach reflects real-world reimbursement review processes, which involve both clinical and administrative expertise.

### LLM Evaluation

We evaluated 6 LLMs from 3 providers: Gemini 3.1 Pro (gemini-3.1-pro-preview) and Gemini 3 Flash (gemini-3-flash-preview) from Google; Claude Opus 4.6 (claude-opus-4‐6) and Claude Sonnet 4.6 (claude-sonnet-4‐6) from Anthropic; and GPT-5.4 (gpt-5.4-2026-03-05) and GPT-5 Mini (gpt-5-mini-2025-08-07) from OpenAI. All models were evaluated under the primary condition, in which the original HIRA guideline PDF was provided via each model’s native document upload feature. Guideline documents were provided as 3 cancer-specific PDF files corresponding to cervical, uterine, and ovarian cancer. Because the set of user-adjustable hyperparameters differed across models and the evaluation included both reasoning and nonreasoning models, all models were evaluated using provider-default settings, and no parameter was explicitly modified. To assess response stability, each model was run 3 times under the same condition.

All models received the same standardized prompt template (Textbox 1). The system prompt instructed the model to act as an expert reviewer of Korean NHI reimbursement for oncology regimens and to return a structured JSON response containing a 3-class decision (eligible, ineligible, or undeterminable) and a single-sentence rationale. The user prompt specified the cancer type, regimen name, and structured clinical and administrative attributes.

Textbox 1. **Standardized prompt template for reimbursement eligibility determination.**

**System Prompt:**

You are an expert reviewer of Korean National Health Insurance reimbursement for oncology regimens.

Given the clinical and administrative attributes and any provided material, decide whether the named regimen is reimbursable.

Respond with exactly one JSON object using this schema:

{“decision”:“eligible|ineligible|undeterminable,”“reason”:“<one short sentence>”}

The following are the outcome definitions:

- Eligible: All required conditions are met.
- Ineligible: 1 or more conditions are explicitly not met, regardless of whether other conditions are unevaluable. The condition is present but violates the criterion.
- Undeterminable: No condition is explicitly not met, but 1 or more conditions are unevaluable due to absent clinical information. Because no criterion is violated, the case cannot be classified as ineligible, yet a determination cannot be reached.

**User Prompt:**

Cancer type:<cancer_type>

Regimen:<regimen_name>

Clinical and administrative attributes:<structured attributes>

Task: Determine whether this patient is eligible for reimbursement for the regimen above.

### Statistical Analysis

Each model was evaluated over 3 independent runs. To capture run-to-run consistency of the models, accuracy was recorded to compute mean and SD. A final predicted label for each case was determined by majority voting across the 3 runs. A 3-way tie was resolved as undeterminable, reflecting the model’s own uncertainty about the case.

All evaluation metrics—precision, recall, and *F*_1_-score—were calculated from these majority-voted predictions. Ninety-five percent CIs for accuracy, precision, and recall were calculated by Wilson method, and those for the *F*_1_-score were obtained by bootstrapping (n=1000). The Bhapkar test of marginal homogeneity was used to assess whether the predicted outcome distribution differed from the observed distribution across the 3 outcome classes. Analyses were performed using Python 3.10 with SciPy.

### Error Analysis

We further conducted a retrospective error analysis of all misclassified cases to characterize how models failed across the benchmark. Because the benchmark was explicitly designed around a tristate adjudication framework, model errors could be interpreted according to the adjudication principle they violated.

We therefore conducted a logic-based error analysis in which each expected-predicted outcome pair was mapped to an error category. Representative rationales and guideline locations were then reviewed to characterize recurrent structural sources of the dominant error category. This rationale review was performed blinded to model identity to mitigate potential reviewer bias.

### Sensitivity Analysis

Sensitivity analysis consisted of 3 strategies. First, to examine the effect of input format, the guideline file (PDF) was parsed into structured text (Markdown) using the pdfplumber library, and all models were evaluated with the extracted text as input. Second, to assess the effect of information access, search-enabled conditions were evaluated on all models. This setting was intended to reflect scenarios in which patients or health care providers use LLMs with web search enabled. Third, to investigate the effect of prompting strategy, a prompt informed by the error analysis findings was evaluated, instructing models to verify each eligibility condition step by step.

For the sensitivity analysis, each prediction was dichotomized as correct or incorrect against the reference standard. Pairwise comparisons of correctness between the baseline and each sensitivity condition were then performed using McNemar test, with Benjamini-Hochberg correction for the false discovery rate. Adjusted *P* values are reported as *q* values.

### Ethical Considerations

This study did not involve human participants, real patient data, or protected health information. All patient scenarios were synthetically constructed based on publicly available reimbursement guidelines published by the HIRA in South Korea.

## Results

### Benchmark Statistics

Agreement between the utilization review nurse and gynecologic oncology experts was initially 95% (211/222; Cohen κ=0.93). Eleven cases were identified in which specific qualifying conditions had not been fully reflected in the benchmark scenarios. These discrepancies were resolved through consensus while preserving the balanced class structure.

The final benchmark composition is summarized in Table 2. A total of 74 anticancer regimens (15 cervical, 17 uterine, and 42 ovarian) were each assigned 3 outcome scenarios, yielding 222 cases. The mean number of eligibility conditions per regimen was 4.2 (SD 1.9; range 2‐11). Uterine (mean 4.5, SD 2.8; range 3-11) and ovarian cancer regimens showed comparable mean complexity (mean 4.5, SD 1.3; range 2-8 for both), but uterine cancer included the most complex individual regimens, with up to 11 eligibility conditions. Cervical cancer had the simplest criteria (mean 2.9, SD 1.4; range 2‐6).

**Table 2.** Benchmark composition by cancer type (N=222).

Characteristic	Cervical	Uterine	Ovarian	Total
Regimens, n	15	17	42	74
Benchmark cases, n	45	51	126	222
Unique clinical attribute types, n	10	14	24	38^a^
Conditions per regimen, mean (SD; range)	2.9 (1.4; 2-6)	4.5 (2.8; 3-11)	4.5 (1.3; 2-8)	4.2 (1.9; 2-11)

a The total reflects the number of distinct attribute types after deduplication across cancer types.

The upper range of complexity in uterine cancer was largely driven by immunotherapy regimens such as pembrolizumab plus lenvatinib, which required up to 11 simultaneous conditions, including deficient mismatch repair or microsatellite instability-high status, Eastern Cooperative Oncology Group performance score, and prior treatment history.

### Overall LLM Performance

Overall accuracy ranged from 77.9% (GPT-5.4) to 88.7% (Gemini 3.1 Pro) across the 6 models. No malformed outputs were observed. Performance was relatively stable across runs, with SDs ranging from 0.3 to 2.0 percentage points (pp). Despite these differences in overall accuracy, all models showed the same general pattern of substantially lower recall for undeterminable cases than for eligible or ineligible cases. Three-way ties across runs were rare, occurring in only 1 case out of all model evaluations (222 cases × 6 models). Detailed results are provided in Multimedia Appendix 1.

### Analysis by Outcome Class

**Figure 3.**
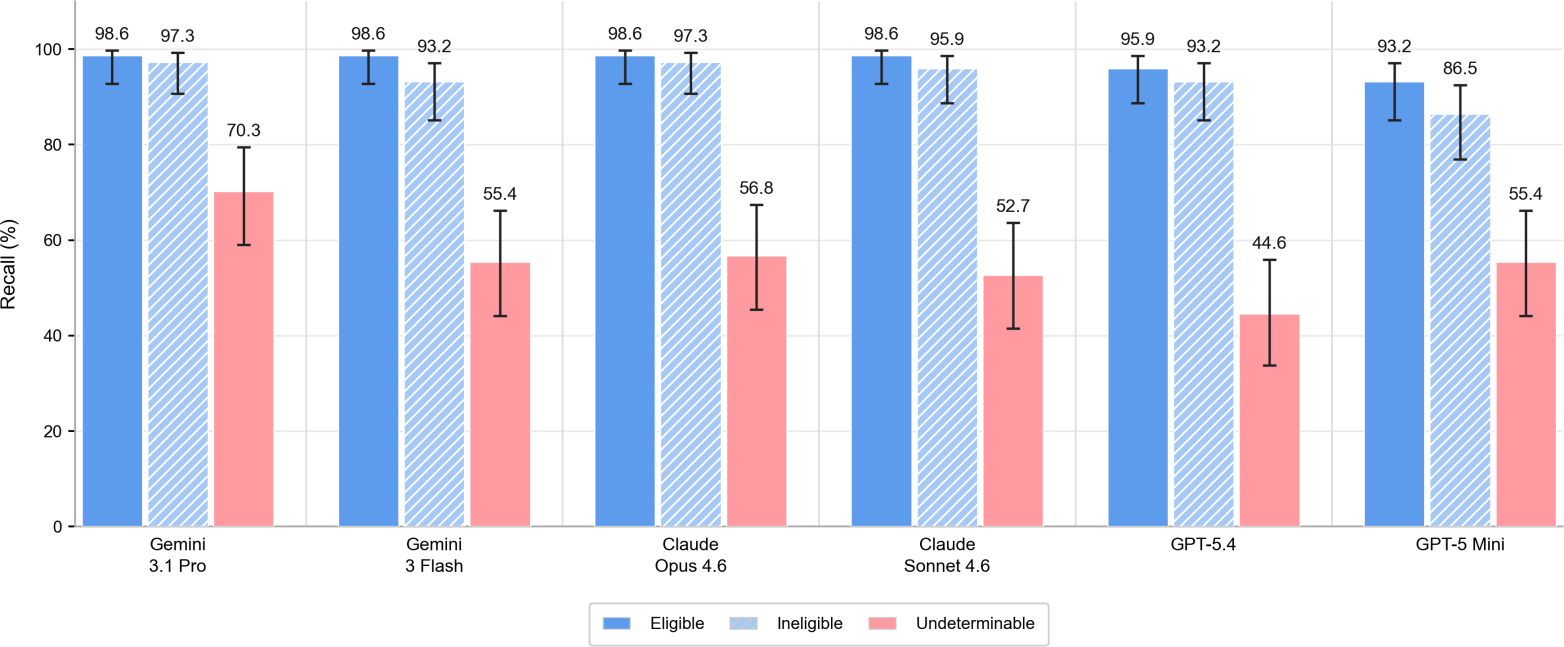
Recall by model with 95% CI (n=74 per class, majority vote). Eligible (solid blue) cases met all required conditions. Ineligible (hatched blue) cases contained at least 1 explicitly not-met condition. Undeterminable (red) cases contained no explicitly not-met condition but at least 1 unevaluable condition due to missing information.

The LLM output was analyzed by 3 outcome classes (Figure 3). For eligible cases, recall was high across models (93.2%‐98.6%), and ineligible cases showed similarly strong performance (86.5%‐97.3%). By contrast, undeterminable cases showed a marked decline across all models, ranging from 44.6% to 70.3%. Among the models, Gemini 3.1 Pro showed the highest recall for undeterminable cases (70.3%), whereas GPT-5.4 showed the lowest (44.6%). The full results with precision, recall, *F*_1_-score, and confusion matrix are provided in Multimedia Appendix 2.

Analysis of misclassification direction for undeterminable cases (Table 3, 44.6%‐70.3%) revealed that models predominantly misclassified these cases as eligible (28.4%‐50%) rather than ineligible (0%‐5.4%). This pattern was observed across all 6 models. Consistent with this pattern, Bhapkar tests showed significant marginal heterogeneity between observed and predicted outcome distributions for all models (all *P*<.001; Multimedia Appendix 3).

**Table 3.** Misclassification direction for undeterminable cases by model (majority vote, n=74 per model).

Model	Predicted eligible, n (%)	Predicted ineligible, n (%)	Predicted undeterminable, n (%)
Gemini 3.1 Pro	21 (28.4)	1 (1.4)	52 (70.3)
Gemini 3 Flash	33 (44.6)	0 (0)	41 (55.4)
Claude Opus 4.6	31 (41.9)	1 (1.4)	42 (56.8)
Claude Sonnet 4.6	35 (47.3)	0 (0)	39 (52.7)
GPT-5.4	37 (50)	4 (5.4)	33 (44.6)
GPT-5 Mini	31 (41.9)	2 (2.7)	41 (55.4)

### Analysis by Cancer Type

Performance varied across cancer types. Cervical cancer showed the highest mean recall across 6 models (92.6%), followed by ovarian (82.9%) and uterine (71.9%) cancer. Per-regimen complexity was lowest for cervical cancer (mean 2.9, SD 1.4; range 2‐6 conditions) and highest for uterine cancer (mean 4.5, SD 2.8; range 3‐11). Notably, recall varied substantially by outcome class within each cancer type (Table 4). While eligible and ineligible recall remained high across cancer types (89.7%‐100%), undeterminable recall dropped sharply, particularly for uterine cancer (mean 16.7%), where per-regimen complexity was highest. Model-wise results by cancer type and outcome class are provided in Multimedia Appendix 4.

**Table 4.** Pooled recall across cancer types and outcome classes with 95% CI (6 models).

Cancer type	Eligible, % (95% CI)	Ineligible, % (95% CI)	Undeterminable, % (95% CI)	Overall, % (95% CI)
Cervical	100 (95.9‐100.0)	98.9 (94.0‐99.8)	78.9 (69.4‐86.0)	92.6 (88.8‐95.2)
Uterine	99 (94.7‐99.8)	100 (96.4‐100.0)	16.7 (10.7‐25.1)	71.9 (66.6‐76.6)
Ovarian	95.6 (92.4‐97.5)	89.7 (85.3‐92.9)	63.5 (57.4‐69.2)	82.9 (80.1‐85.5)

### Error Analysis

The tristate benchmark design enabled a logic-based characterization of model errors. All 235 misclassified cases were assigned to 1 of 3 error types according to how each misclassification violated the adjudication logic; predicted outcomes and single-sentence rationales were reviewed to characterize each category (Table 5).

**Table 5.** Error categories enabled by the tristate adjudication framework.

Error type and expected outcome	Predicted outcome	Explanation
Information gap-filling		
Undeterminable	Eligible	One or more conditions were not provided, but the model assumed a met value for the missing attribute.
Undeterminable	Ineligible	One or more conditions were not provided, but the model assumed a not-met value for the missing attribute.
Criterion misapplication		
Eligible	Ineligible	All conditions were met, but the model applied a restriction not present in the guideline.
Ineligible	Eligible	One or more conditions were explicitly not met, but the model confirmed eligibility based on the remaining met conditions.
False uncertainty		
Eligible	Undeterminable	All conditions were met, but the model treated one or more conditions as unevaluable and withheld judgment.
Ineligible	Undeterminable	One or more conditions were explicitly not met, but the model prioritized an unevaluable condition over the not-met finding.

The most common error was information gap-filling (196/235, 83.4%), in which models inferred a definitive outcome from incomplete scenarios rather than recognizing missing information. The second most common error type was criterion misapplication (n=20, 8.5%), in which models classified cases as eligible despite 1 or more clearly not-met conditions or, conversely, classified cases as ineligible despite all conditions being met. Third, false uncertainty (n=19, 8.1%) refers to cases where models inferred undeterminable despite sufficient information to adjudicate eligible or ineligible. This pattern occurred most frequently in ineligible scenarios where 1 or more conditions were unevaluable but at least 1 clearly unmet condition was present.

To further understand the dominant error type, information gap cases were analyzed in relation to guideline structure. Reimbursement guidelines are organized hierarchically (Textbox 2), and model performance varied with structural complexity. In particular, subheading (L2) and footnote (L4) were the weak points of the models.

Textbox 2. **Example structure of anticancer drug eligibility guideline.**

[L1] Global Caveat: Cancer-type-level conditions applied to all regimens (eg, default scope of covered indications)

[L2] Treatment Intent A

  └─ Table

  └─[L3] Regimen A [Indication A]

  └─[L3] Regimen B [Indication B]

  └─…

[L2] Treatment Intent B

  └─ Table

  └─[L3] Regimen A [Indication C]

  └─[L3] Regimen C [Indication D]

  └─…

[L2] Treatment Intent C

  └─[L3] Regimen D [Indication E]

  └─[L3] Regimen E [Indication F]

  └─…

[L2] Treatment Intent D

  └─[L3] Regimen F [Indication G]*

  └─….

[L4] *Footnote: Externalized condition at document tail, applied via explicit cross-reference (eg, “see Note 1”)

The largest subtype involved models ignoring the document subheading (L2) that specifies therapeutic intent (eg, palliative, maintenance). This distinction is clinically significant because identical regimens may appear under multiple therapeutic purposes with different indications, and reimbursement is granted only for the purpose under which the regimen is listed.

Another subtype involved footnote (L4) recognition. Designated institution requirement for immune checkpoint inhibitor, restricting eligible prescribing sites to those with qualified specialist staffing, appeared only in guideline footnotes, and most models systematically missed this condition.

### Sensitivity Analysis

Building on the failure modes identified in the error analysis, 3 sensitivity conditions were evaluated (Multimedia Appendix 5). These analyses included structured text, web search–enabled, and structure-guided prompt conditions.

In the structured text condition, the parsed guideline in Markdown format was provided to the models. Performance declined across all 6 models, with statistically significant degradation observed in the 2 Claude models (Claude Opus 4.6: Δ=−10.4 pp; Claude Sonnet 4.6: Δ=−10.8 pp; both *q*<.001). The remaining 4 models showed smaller declines that did not reach significance.

The web search–enabled condition assessed the effect of additional information access alongside the guideline document, while the structure-guided prompt condition incorporated the hierarchical levels of the guideline (Textbox 2) into the prompt and instructed models to verify each eligibility condition step by step against the corresponding level. Neither condition produced a statistically significant change from baseline in any model (all *q*>.05).

Notably, web search tool invocation rates ranged from 0% to 3.2% across the 6 models under the web search–enabled condition. Token usage nevertheless increased substantially: OpenAI models showed approximately 4500 additional input tokens per case from the embedded tool specification, while Claude models produced longer reasoning.

## Discussion

### Principal Results

This study developed a benchmark for anticancer drug reimbursement eligibility determination covering 3 gynecologic cancers and used it to evaluate 6 LLMs from 3 providers. The benchmark was based on a tristate adjudication framework that distinguishes condition-level states (met, not met, unevaluable) and case-level outcomes (eligible, ineligible, and undeterminable) for eligibility verification. Case-level outcomes are derived from the aggregation of condition-level states.

The principal findings are threefold. First, overall accuracy varied across models, ranging from 77.9% to 88.7%, indicating broadly comparable performance across the 6 evaluated models. Second, performance varied sharply by outcome class. While eligible and ineligible cases were classified with consistently high recall, undeterminable cases posed the greatest challenge across all models, revealing a consistent limitation to recognize when clinical information is insufficient for a determination. Third, the tristate benchmark design enabled a logic-based characterization of model errors. Rather than treating all incorrect predictions as equivalent, the framework distinguished information gap-filling, criterion misapplication, and false uncertainty according to how each misclassification violated the expected adjudication logic.

These findings suggest that LLMs may help reduce the manual burden of eligibility review for clearly eligible and ineligible cases. However, these models should be positioned as decision-support tools operating under human oversight, not as autonomous decision-makers. The systematic failure in undeterminable cases represents the most clinically consequential limitation, as models tend to resolve ambiguity toward eligibility rather than deferring judgment when information is incomplete.

The dominant undeterminable failure pattern—information gap-filling—has direct clinical implications. When models misclassified undeterminable cases, the predominant direction was toward eligible rather than ineligible, indicating a tendency to infer eligibility from incomplete information. The severity of this pattern varied by cancer type, corresponding to the number of eligibility conditions per regimen. Uterine cancer regimens, which had the largest mean number of eligibility conditions, showed the lowest undeterminable recall.

This tendency has potential financial implications for hospitals. Under South Korea’s retrospective claims review structure, ineligibility identified during postadministration audit can result in full denial of previously reimbursed drug costs, with the burden shifted to the prescribing institution. Because high-cost agents such as immune checkpoint inhibitors, antiangiogenic agents, and poly (ADP-ribose) polymerase inhibitors are reimbursed under exception-coverage provisions for severe diseases, even a small number of misclassifications can accumulate into substantial losses. Given that per-cycle costs typically range from KRW 3‐6 million (approximately US $2200‐$4400), cumulative nonrecoverable costs can reach tens of millions of KRW per patient. Although the per-case inference cost itself is minimal (approximately US $0.002‐$0.10 across the 6 models), an erroneous determination at this cost can still translate into substantial financial losses through retrospective denial.

Meanwhile, sensitivity analysis revealed 3 patterns. First, converting the guideline file to structured text degraded performance across all models, indicating that document structure conveys eligibility logic lost during text extraction. Second, enabling web search did not produce a statistically significant change from baseline for any model (all *q*>.05), with models defaulting to the provided guideline rather than calling the search tool. This pattern indicates that in realistic scenarios where authoritative source documents are accessible to the LLM, the models rely on the provided context, and enabling the search tool neither improved nor degraded outcomes while increasing inference cost. Third, structure-guided prompting did not produce a statistically significant improvement for any of the 6 models (all *q*>.05), indicating that explicit structural guidance combined with step-by-step per-condition verification was insufficient to overcome the inherent limitations in hierarchical guideline reasoning observed in this benchmark.

Taken together, these results indicate that current LLMs are not yet reliable as standalone tools for reimbursement eligibility determination, particularly when clinical information is incomplete. Because neither input reformatting, information augmentation, nor prompt modification alone reliably improved performance, more systematic integration strategies will be needed to deploy LLMs safely in reimbursement workflows.

### Comparison With Prior Work

Most LLM benchmarks in the medical domain have focused on knowledge assessment through multiple-choice examinations such as MedQA [21], while real-world task evaluation remains scarce [22]. Recent frameworks such as MedHELM [23] have begun addressing this gap but reveal that administrative and workflow tasks remain the weakest evaluation category.

Within guideline-based reasoning, MedGUIDE [24] and CPGPrompt [25] evaluated LLM adherence to cancer treatment decision trees and referral classification guidelines, respectively, both finding that models frequently deviate from structured conditional logic. Outside the medical domain, RuleArena [26] reported similar difficulties with complex rule-guided reasoning. However, none of these benchmarks test whether models can recognize when information is insufficient for a determination; all evaluate scenarios in which sufficient information is provided and a definitive answer exists.

This limitation is directly relevant to our findings. Recent abstention studies—AbstentionBench [27] across 20 datasets and MedAbstain [28] in medical question answering—showed that providing an explicit abstention option consistently increased model uncertainty and safer abstention behavior, whereas scaling model size or applying structure-guided prompting yielded little improvement.

Our undeterminable outcome class parallels these abstention scenarios in a domain-specific context. However, whereas these benchmarks test whether models can detect missing information within a self-contained question, our benchmark additionally requires models to extract eligibility conditions from a hierarchically structured guideline document and then determine whether the patient scenario provides sufficient information to evaluate each condition.

To our knowledge, no prior benchmark has evaluated LLM performance on health insurance reimbursement eligibility. This study addresses this gap by introducing a benchmark grounded in national anticancer drug coverage criteria with expert validation, incorporating an undeterminable outcome class that explicitly tests recognition of incomplete information.

### Limitations

This study has several limitations. First, the benchmark was limited to 3 gynecologic cancers based on South Korean NHI guidelines and relied on a relatively small dataset. Its generalizability to other cancer types, guideline versions, or insurance systems requires further validation.

Second, all patient scenarios were synthetically constructed in a structured format. This design does not capture the additional uncertainty introduced by extracting relevant attributes from unstructured records. Because real-world clinical documentation is often incomplete and unstructured, the performance observed in this study may overestimate model utility in practice.

Third, some eligibility conditions appeared in the global caveat and footnotes; errors involving these structural elements may partly reflect document representation rather than reasoning limitations of models alone.

Fourth, our benchmark adopts a strict rule that treats any missing information required by guideline criteria as undeterminable. This reflects a fundamental distinction between clinical reasoning and administrative reasoning. For instance, the absence of a prior treatment record may suggest a patient who has not previously been treated in clinical reasoning but constitutes missing evidence in administrative adjudication. Findings should, therefore, be interpreted as specific to administrative reasoning tasks.

Fifth, while the qualitative error analysis was conducted with model identities blinded, LLM outputs often contain stylistic fingerprints (eg, characteristic phrasing or cadences) that may make perfect blinding difficult. Residual unblinding may, therefore, introduce some bias into our error categorization.

Finally, models were tested with provider-default settings without fine-tuning or agentic frameworks, which may underestimate achievable performance.

### Future Work

Several directions arise from these limitations. Future work should expand the benchmark to additional cancer types and guideline versions and validate it using real clinical documentation to better reflect performance under unstructured and incomplete records. A direct human performance baseline, in which experts solve the same cases under identical conditions, would also be valuable to contextualize LLM accuracy against expert-level performance.

Methodologically, agentic approaches that iteratively verify each condition against the guideline may improve accuracy on complex hierarchical structures. In addition, hybrid neuro-symbolic architectures that pair LLM-based understanding with structured rule engines have shown initial promise in adjacent health care tasks [29]; their applicability to reimbursement reasoning under incomplete information, particularly in handling rigid AND/OR logical constraints, remains to be evaluated.

### Conclusion

The tristate adjudication framework introduced in this study offers a distinctive approach to evaluating LLM behavior in logical reasoning contexts characterized by variable information completeness. In this benchmark, LLMs classified clearly eligible and ineligible cases with relatively high recall but showed limited reliability on undeterminable cases. The dominant error pattern was information gap-filling, in which models tended to infer eligibility rather than withhold judgment. These findings indicate that LLMs, in their current form, should be deployed as supervised decision-support tools rather than as independent adjudicators in reimbursement review.

## Supplementary material

10.2196/95877 Multimedia Appendix 1 Overall accuracy across repeated runs and majority-voted with 95% CI.

10.2196/95877 Multimedia Appendix 2 Large language model performance per class with majority voting (n=74 per class; 95% CI).

10.2196/95877 Multimedia Appendix 3 Bhapkar test of marginal homogeneity comparing each large language model's predicted distribution against the observed distribution (eligible=74, ineligible=74, undeterminable =74; balanced design) across the 3 eligibility categories.

10.2196/95877 Multimedia Appendix 4 Model-wise recall by cancer type and outcome class with 95% CI.

10.2196/95877 Multimedia Appendix 5 Sensitivity analysis results with pairwise comparisons against baseline using McNemar test with Benjamini-Hochberg correction.

10.2196/95877 Checklist 1 TRIPOD-LLM checklist.

## Abbreviations

HIRA: Health Insurance Review & Assessment Service
LLM: large language model
NHI: National Health Insurance
pp: percentage points
TRIPOD-LLM: Transparent Reporting of a Multivariable Prediction Model for Individual Prognosis or Diagnosis-Large Language Model

## References

[1] Jiwani A, Himmelstein D, Woolhandler S, Kahn JG (2014). Billing and insurance-related administrative costs in United States’ health care: synthesis of micro-costing evidence. BMC Health Serv Res.

[2] Tseng P, Kaplan RS, Richman BD, Shah MA, Schulman KA (2018). Administrative costs associated with physician billing and insurance-related activities at an academic health care system. JAMA.

[3] Erickson SM, Rockwern B, Koltov M, McLean RM, Medical Practice and Quality Committee of the American College of Physicians (2017). Putting patients first by reducing administrative tasks in health care: a position paper of the American College of Physicians. Ann Intern Med.

[4] Kyle MA, Feng KY, Wade CG, Yaver M (2025). Patient administrative burden: a scoping review. Health Aff Sch.

[5] Richman BD, Kaplan RS, Kohli J (2022). Billing and insurance-related administrative costs: a cross-national analysis. Health Aff (Millwood).

[6] Sohn M, Jung M (2016). Effects of public and private health insurance on medical service utilization in the National Health Insurance System: national panel study in the Republic of Korea. BMC Health Serv Res.

[7] Kim JA, Yoon S, Kim LY, Kim DS (2017). Towards actualizing the value potential of Korea Health Insurance Review and Assessment (HIRA) data as a resource for health research: strengths, limitations, applications, and strategies for optimal use of HIRA data. J Korean Med Sci.

[8] Kim L, Kim JA, Kim S (2014). A guide for the utilization of Health Insurance Review and Assessment Service National Patient Samples. Epidemiol Health.

[9] Shin HC, Park YT, Lee YT, Jo EC (2015). Healthcare utilization monitoring system in Korea. Healthc Inform Res.

[10] Yun J, Chang Y, Jo M, Heo Y, Kim DS (2025). National expenditures on anticancer and immunomodulating agents during 2013-2022 in Korea. J Korean Med Sci.

[11] (2026). Cancer drug and regimen guidelines: public announcements [Report in Korean]. https://www.hira.or.kr/bbsDummy.do?pgmid=HIRAA030023010000.

[12] (2026). AI as a healthcare ally: how Americans are navigating the system with ChatGPT. https://cdn.openai.com/pdf/2cb29276-68cd-4ec6-a5f4-c01c5e7a36e9/OpenAI-AI-as-a-Healthcare-Ally-Jan-2026.pdf.

[13] Seo J, Choi D, Kim T (2024). Evaluation framework of large language models in medical documentation: development and usability study. J Med Internet Res.

[14] Song JW, Park J, Kim JH, You SC (2025). Large language model assistant for emergency department discharge documentation. JAMA Netw Open.

[15] Jones MD, Torgbi M, Tayyar Madabushi H (2026). Improving the understandability of clinical guidelines: development and evaluation of a GPT-4-based pipeline. J Med Internet Res.

[16] Zheng NS, Keloth VK, You K (2025). Detection of gastrointestinal bleeding with large language models to aid quality improvement and appropriate reimbursement. Gastroenterology.

[17] Hou Z, Liu H, Bian J, He X, Zhuang Y (2025). Enhancing medical coding efficiency through domain-specific fine-tuned large language models. Npj Health Syst.

[18] Smith PC, Araya-Guerra R, Bublitz C (2005). Missing clinical information during primary care visits. JAMA.

[19] Burnett SJ, Deelchand V, Franklin BD, Moorthy K, Vincent C (2011). Missing clinical information in NHS hospital outpatient clinics: prevalence, causes and effects on patient care. BMC Health Serv Res.

[20] Gallifant J, Afshar M, Ameen S (2025). The TRIPOD-LLM reporting guideline for studies using large language models. Nat Med.

[21] Jin D, Pan E, Oufattole N, Weng WH, Fang H, Szolovits P (2021). What disease does this patient have? A large-scale open domain question answering dataset from medical exams. Appl Sci.

[22] Bedi S, Liu Y, Orr-Ewing L (2025). Testing and evaluation of health care applications of large language models: a systematic review. JAMA.

[23] Bedi S, Cui H, Fuentes M (2026). Holistic evaluation of large language models for medical tasks with MedHELM. Nat Med.

[24] Li X, Gao M, Hao Y MedGUIDE: benchmarking clinical decision-making in large language models. https://openreview.net/pdf/07c2a85f2355803fe92312f6a9808c0e5065ddef.pdf.

[25] Deng R, Martin G, Wang T (2026). CPGPrompt: translating clinical guidelines into large language model-executable decision support. J Am Med Inform Assoc.

[26] Zhou R, Hua W, Pan L RuleArena: a benchmark for rule-guided reasoning with LLMs in real-world scenarios.

[27] Kirichenko P, Ibrahim M, Chaudhuri K, Bell SJ AbstentionBench: reasoning llms fail on unanswerable questions. https://proceedings.neurips.cc/paper_files/paper/2025/hash/fb122bfc3f0127a94ded048b5b03496f-Abstract-Datasets_and_Benchmarks_Track.html.

[28] Machcha S, Yerra S, Gupta S Knowing when to abstain: medical LLMs under clinical uncertainty.

[29] Prenosil GA, Weitzel TK, Bello SC (2025). Neuro-symbolic AI for auditable cognitive information extraction from medical reports. Commun Med (Lond).

[30] Seo-see/k-NHIB. GitHub.

[31] K-NHIB: Korean National Health Insurance Benchmark. Zenodo.

