# Supplementary material for: Assessing Eligibility for Anticancer Drug Health Insurance Reimbursement Using Large Language Models: Benchmark Development and Comparative Study
Source: J Med Internet Res. 2026 Jun 15;28:e95877. doi: 10.2196/95877 (PMC13268259; doi:10.2196/95877)
Supplement: Multimedia Appendix 1 [file jmir-v28-e95877-s001.docx]

Multimedia Appendix 1. Overall accuracy across repeated runs and majority-voted with 95% CI.

| Model | R1 Accuracy (%) | R2 Accuracy (%) | R3 Accuracy (%) | Mean ± SD | MV Accuracy (%) |
| --- | --- | --- | --- | --- | --- |
| Claude Opus 4.6 | 83.8 | 83.8 | 83.3 | 83.6 ± 0.3 | 84.2 [78.9–88.4] |
| Claude Sonnet 4.6 | 83.8 | 82.0 | 80.2 | 82.0 ± 1.8 | 82.4 [76.9–86.9] |
| Gemini 3.1 Pro | 87.8 | 89.2 | 88.3 | 88.4 ± 0.7 | 88.7 [83.9–92.3] |
| Gemini 3 Flash | 82.0 | 82.9 | 82.4 | 82.4 ± 0.5 | 82.4 [76.9–86.9] |
| GPT-5.4 | 78.4 | 78.4 | 77.0 | 77.9 ± 0.8 | 77.9 [72.0–82.9] |
| GPT-5 Mini | 75.7 | 79.3 | 78.8 | 77.9 ± 2.0 | 78.4 [72.5–83.3] |
