# Supplementary material for: Assessing Eligibility for Anticancer Drug Health Insurance Reimbursement Using Large Language Models: Benchmark Development and Comparative Study
Source: J Med Internet Res. 2026 Jun 15;28:e95877. doi: 10.2196/95877 (PMC13268259; doi:10.2196/95877)

Multimedia Appendix 2. Large language model performance per class with majority voting (n=74 per class; 95% CI).

a. Eligible class

| Model | Precision (%) | Recall (%) | *F*_1_-score (%) |
| --- | --- | --- | --- |
| Gemini 3.1 Pro | 77.7 [68.2–84.9] | 98.6 [92.7–99.8] | 86.9 [80.9–92.0] |
| Gemini 3 Flash | 68.2 [58.9–76.3] | 98.6 [92.7–99.8] | 80.7 [74.0–86.5] |
| Claude Opus 4.6 | 69.5 [60.2–77.5] | 98.6 [92.7–99.8] | 81.6 [75.1–87.3] |
| Claude Sonnet 4.6 | 67.0 [57.7–75.1] | 98.6 [92.7–99.8] | 79.8 [73.0–85.7] |
| GPT-5.4 | 63.4 [54.2–71.7] | 95.9 [88.7–98.6] | 76.3 [69.0–82.7] |
| GPT-5 Mini | 66.3 [56.8–74.7] | 93.2 [85.1–97.1] | 77.5 [70.3–83.8] |

b. Ineligible class

| Model | Precision (%) | Recall (%) | *F*_1_-score (%) |
| --- | --- | --- | --- |
| Gemini 3.1 Pro | 97.3 [90.7–99.3] | 97.3 [90.7–99.3] | 97.3 [94.2–99.4] |
| Gemini 3 Flash | 98.6 [92.3–99.7] | 93.2 [85.1–97.1] | 95.8 [92.2–98.7] |
| Claude Opus 4.6 | 97.3 [90.7–99.3] | 97.3 [90.7–99.3] | 97.3 [94.4–99.4] |
| Claude Sonnet 4.6 | 98.6 [92.5–99.8] | 95.9 [88.7–98.6] | 97.3 [94.3–99.4] |
| GPT-5.4 | 92.0 [83.6–96.3] | 93.2 [85.1–97.1] | 92.6 [87.9–96.5] |
| GPT-5 Mini | 92.8 [84.1–96.9] | 86.5 [76.9–92.5] | 89.5 [83.7–94.4] |

c. Undeterminable class

| Model | Precision (%) | Recall (%) | *F*_1_-score (%) |
| --- | --- | --- | --- |
| Gemini 3.1 Pro | 96.3 [87.5–99.0] | 70.3 [59.1–79.5] | 81.2 [73.2–88.0] |
| Gemini 3 Flash | 91.1 [79.3–96.5] | 55.4 [44.1–66.2] | 68.9 [58.5–77.8] |
| Claude Opus 4.6 | 97.7 [87.9–99.6] | 56.8 [45.4–67.4] | 71.8 [61.9–80.3] |
| Claude Sonnet 4.6 | 95.1 [83.9–98.7] | 52.7 [41.5–63.7] | 67.8 [57.1–77.0] |
| GPT-5.4 | 94.3 [81.4–98.4] | 44.6 [33.8–55.9] | 60.6 [48.5–70.7] |
| GPT-5 Mini | 83.7 [71.0–91.5] | 55.4 [44.1–66.2] | 66.7 [56.4–75.8] |

d. Confusion matrix


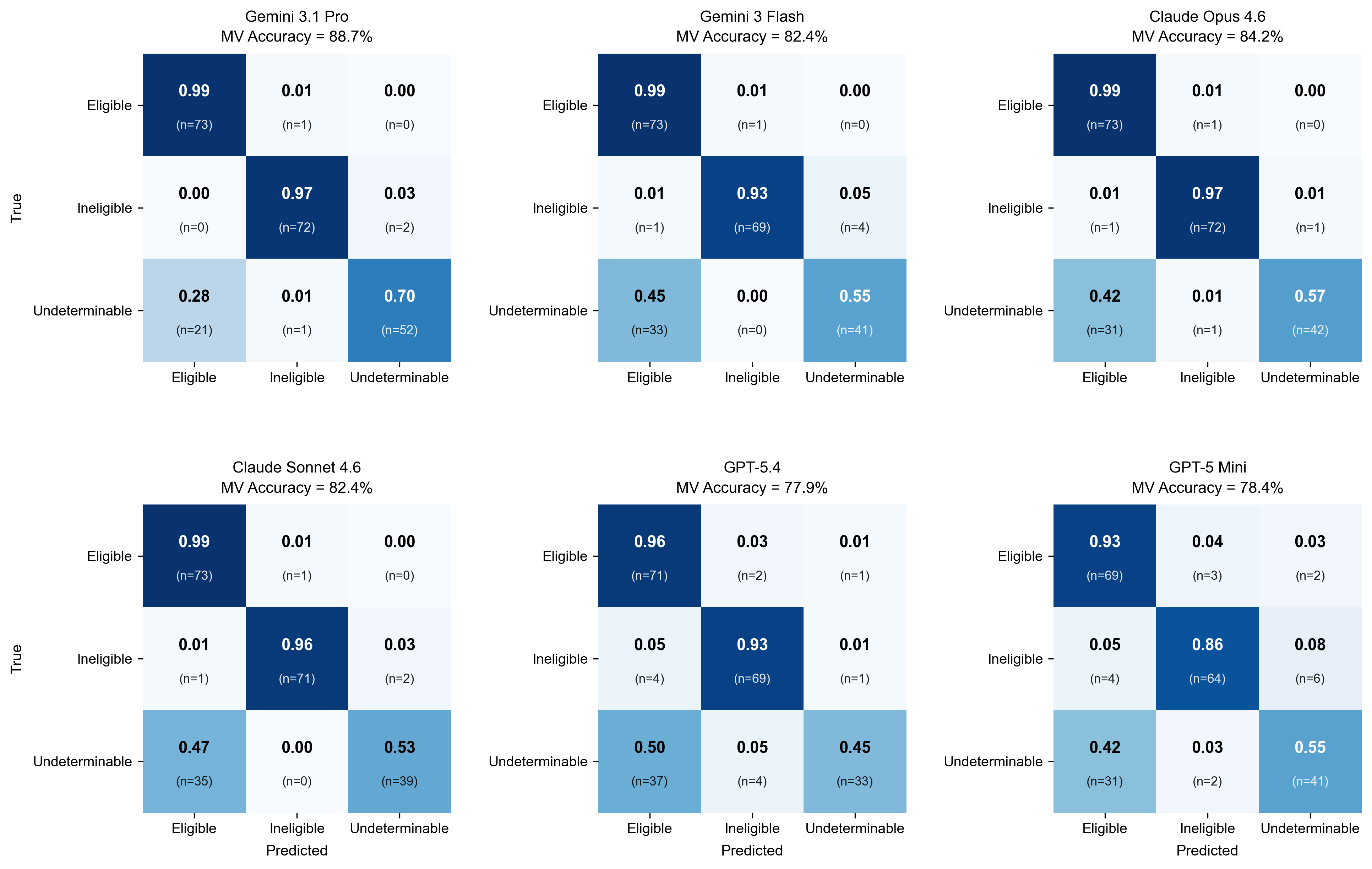

Supplement: Multimedia Appendix 2 [file jmir-v28-e95877-s002.docx]
