# Supplementary material for: Assessing Eligibility for Anticancer Drug Health Insurance Reimbursement Using Large Language Models: Benchmark Development and Comparative Study
Source: J Med Internet Res. 2026 Jun 15;28:e95877. doi: 10.2196/95877 (PMC13268259; doi:10.2196/95877)
Supplement: Multimedia Appendix 3 [file jmir-v28-e95877-s003.docx]

Multimedia Appendix 3. Bhapkar test of marginal homogeneity comparing each large language model’s predicted distribution against the observed distribution (eligible=74, ineligible=74, undeterminable=74; balanced design) across the 3 eligibility categories.

| Model | Predicted Eligible, n | Predicted Ineligible, n | Predicted Undeterminable, n | T (Bhapkar) | df | *P* value |
| --- | --- | --- | --- | --- | --- | --- |
| Claude Opus 4.6 | 105 | 74 | 43 | 31.03 | 2 | <.001 |
| Claude Sonnet 4.6 | 109 | 72 | 41 | 33.11 | 2 | <.001 |
| Gemini 3.1 Pro | 94 | 74 | 54 | 18.39 | 2 | <.001 |
| Gemini 3 Flash | 107 | 70 | 45 | 31.87 | 2 | <.001 |
| GPT-5.4 | 112 | 75 | 35 | 36.57 | 2 | <.001 |
| GPT-5 Mini | 104 | 69 | 49 | 22.50 | 2 | <.001 |
