# Supplementary material for: Assessing Eligibility for Anticancer Drug Health Insurance Reimbursement Using Large Language Models: Benchmark Development and Comparative Study
Source: J Med Internet Res. 2026 Jun 15;28:e95877. doi: 10.2196/95877 (PMC13268259; doi:10.2196/95877)
Supplement: Multimedia Appendix 4 [file jmir-v28-e95877-s004.docx]

Multimedia Appendix 4. Model-wise recall by cancer type and outcome class with 95% CI.

a. Cervical cancer (n=15 cases per outcome class; 45 cases per model)

| Model | Eligible (%) | Ineligible (%) | Undeterminable (%) | Overall (%) |
| --- | --- | --- | --- | --- |
| Claude Opus 4.6 | 100.0 [79.6–100.0] | 100.0 [79.6–100.0] | 86.7 [62.1–96.3] | 95.6 [85.2–98.8] |
| Claude Sonnet 4.6 | 100.0 [79.6–100.0] | 100.0 [79.6–100.0] | 46.7 [24.8–69.9] | 82.2 [68.7–90.7] |
| Gemini 3.1 Pro | 100.0 [79.6–100.0] | 100.0 [79.6–100.0] | 86.7 [62.1–96.3] | 95.6 [85.2–98.8] |
| Gemini 3 Flash | 100.0 [79.6–100.0] | 100.0 [79.6–100.0] | 86.7 [62.1–96.3] | 95.6 [85.2–98.8] |
| GPT-5.4 | 100.0 [79.6–100.0] | 93.3 [70.2–98.8] | 80.0 [54.8–93.0] | 91.1 [79.3–96.5] |
| GPT-5 Mini | 100.0 [79.6–100.0] | 100.0 [79.6–100.0] | 86.7 [62.1–96.3] | 95.6 [85.2–98.8] |

b. Uterine cancer (n=17 cases per outcome class; 51 cases per model)

| Model | Eligible (%) | Ineligible (%) | Undeterminable (%) | Overall (%) |
| --- | --- | --- | --- | --- |
| Claude Opus 4.6 | 100.0 [81.6–100.0] | 100.0 [81.6–100.0] | 17.6 [6.2–41.0] | 72.5 [59.1–82.9] |
| Claude Sonnet 4.6 | 100.0 [81.6–100.0] | 100.0 [81.6–100.0] | 17.6 [6.2–41.0] | 72.5 [59.1–82.9] |
| Gemini 3.1 Pro | 100.0 [81.6–100.0] | 100.0 [81.6–100.0] | 23.5 [9.6–47.3] | 74.5 [61.1–84.5] |
| Gemini 3 Flash | 100.0 [81.6–100.0] | 100.0 [81.6–100.0] | 5.9 [1.0–27.0] | 68.6 [55.0–79.7] |
| GPT-5.4 | 94.1 [73.0–99.0] | 100.0 [81.6–100.0] | 17.6 [6.2–41.0] | 70.6 [57.0–81.3] |
| GPT-5 Mini | 100.0 [81.6–100.0] | 100.0 [81.6–100.0] | 17.6 [6.2–41.0] | 72.5 [59.1–82.9] |

c. Ovarian cancer (n=42 cases per outcome class; 126 cases per model)

| Model | Eligible (%) | Ineligible (%) | Undeterminable (%) | Overall (%) |
| --- | --- | --- | --- | --- |
| Claude Opus 4.6 | 97.6 [87.7–99.6] | 95.2 [84.2–98.7] | 61.9 [46.8–75.0] | 84.9 [77.6–90.1] |
| Claude Sonnet 4.6 | 97.6 [87.7–99.6] | 92.9 [81.0–97.5] | 69.0 [54.0–80.9] | 86.5 [79.5–91.4] |
| Gemini 3.1 Pro | 97.6 [87.7–99.6] | 95.2 [84.2–98.7] | 83.3 [69.4–91.7] | 92.1 [86.0–95.6] |
| Gemini 3 Flash | 97.6 [87.7–99.6] | 88.1 [75.0–94.8] | 64.3 [49.2–77.0] | 83.3 [75.9–88.8] |
| GPT-5.4 | 95.2 [84.2–98.7] | 90.5 [77.9–96.2] | 42.9 [29.1–57.8] | 76.2 [68.0–82.8] |
| GPT-5 Mini | 88.1 [75.0–94.8] | 76.2 [61.5–86.5] | 59.5 [44.5–73.0] | 74.6 [66.4–81.4] |
