# Supplementary material for: Assessing Eligibility for Anticancer Drug Health Insurance Reimbursement Using Large Language Models: Benchmark Development and Comparative Study
Source: J Med Internet Res. 2026 Jun 15;28:e95877. doi: 10.2196/95877 (PMC13268259; doi:10.2196/95877)
Supplement: Multimedia Appendix 5 [file jmir-v28-e95877-s005.docx]

Multimedia Appendix 5. Sensitivity analysis results with pairwise comparisons against baseline using McNemar's test with Benjamini-Hochberg correction.

| Model | Baseline | Structured Text | | Web Search | | Structure-guided | |
| --- | --- | --- | --- | --- | --- | --- | --- |
|  | Accuracy  [95% CI] | Accuracy  [95% CI] | Δ  (*q*) | Accuracy  [95% CI] | Δ  (*q*) | Accuracy  [95% CI] | Δ  (*q*) |
| Gemini 3.1 Pro | 88.7 [83.9–92.3] | 84.7 [79.4–88.8] | −4.1  (.07) | 86.5 [81.4–90.4] | −2.3  (.32) | 90.0 [85.1–93.3] | +1.2  (.77) |
| Gemini 3 Flash | 82.4 [76.9–86.9] | 79.7 [74.0–84.5] | −2.7  (.36) | 82.9 [77.4–87.3] | +0.5  (1.00) | 82.9 [77.4–87.3] | +0.5  (1.00) |
| Claude Opus 4.6 | 84.2 [78.9–88.4] | 73.9 [67.7–79.2] | −10.4  (<.001) | 87.8 [82.9–91.5] | +3.6  (.23) | 81.5 [75.9–86.1] | −2.7  (.33) |
| Claude Sonnet 4.6 | 82.4 [76.9–86.9] | 71.6 [65.4–77.1] | −10.8  (<.001) | 86.9 [81.9–90.7] | +4.5  (.11) | 84.2 [78.9–88.4] | +1.8  (.58) |
| GPT-5.4 | 77.9 [72.0–82.9] | 72.5 [66.3–78.0] | −5.4  (.08) | 76.1 [70.1–81.3] | −1.8  (.58) | 76.7 [70.5–82.0] | −1.2  (.58) |
| GPT-5 Mini | 78.4 [72.5–83.3] | 76.1 [70.1–81.3] | −2.3  (.64) | 76.6 [70.6–81.7] | −1.8  (.64) | 77.0 [71.1–82.1] | −1.4  (.76) |
