# Supplementary material for: Assessing Eligibility for Anticancer Drug Health Insurance Reimbursement Using Large Language Models: Benchmark Development and Comparative Study
Source: J Med Internet Res. 2026 Jun 15;28:e95877. doi: 10.2196/95877 (PMC13268259; doi:10.2196/95877)
Supplement: Checklist 1 [file jmir-v28-e95877-s006.docx]

Multimedia Appendix 1. TRIPOD-LLM Checklist.

**Research design classification:** E (LLM evaluation). **LLM task classification:** C (classification).

*Abbreviations in original checklist:* M = LLM methods; D = de novo LLM development; E = LLM evaluation; H = LLM evaluation in healthcare settings; C = classification; OF = outcome forecasting; QA = long-form question-answering; IR = information retrieval; DG = document generation; SS = summarization and simplification; MT = machine translation.

| **Section** | **Item** | **Checklist Item** | **Research Design** | **LLM Task** | **Note** |
| --- | --- | --- | --- | --- | --- |
| **Title** | | | | | |
| Title | **1** | Identify the study as developing, fine-tuning, and/or evaluating the performance of an LLM, specifying the task, the target population, and the outcome to be predicted. | All | All | Yes. Identifies the study as benchmark development and comparative evaluation of LLMs for anticancer drug reimbursement eligibility. |
| **Abstract** | | | | | |
| Abstract | **2** | See TRIPOD-LLM for Abstracts. | All | All | Yes. Structured Background / Objective / Methods / Results / Conclusions. |
| **Introduction** | | | | | |
| Background | **3a** | Explain the healthcare context / use case (e.g., administrative, diagnostic, therapeutic, clinical workflow) and rationale for developing or evaluating the LLM, including references to existing approaches and models. | All | All | Yes. Administrative use case (BIR costs, Korean NHI reimbursement), with references to existing LLM applications in coding/reimbursement. |
| Background | **3b** | Describe the target population and the intended use of the LLM in the context of the care pathway, including its intended users in current gold standard practices (e.g., healthcare professionals, patients, public, or administrators). | E, H | All | Yes. The benchmark formalizes the scenario in which LLMs assist clinicians or utilization review nurses performing pre-claim eligibility verification |
| Objectives | **4** | Specify the study objectives, including whether the study describes the initial development, fine-tuning, or validation of an LLM (or multiple stages). | All | All | Yes. Benchmark development plus evaluation of pre-trained LLMs (no fine-tuning). |
| **Methods — Data** | | | | | |
| Data | **5a** | Describe the sources of data separately for the training, tuning, and/or evaluation datasets and the rationale for using these data (e.g., web corpora, clinical research/trial data, EHR data, or unknown). | All | All | Yes. HIRA reimbursement review guidelines (Korean NHI). No training/tuning data. |
| Data | **5b** | Describe the relevant data points and provide a quantitative and qualitative description of their distribution and other relevant descriptors of the dataset (e.g., source, languages, countries of origin). | All | All | Yes. 74 regimens, 222 cases, 3 cancer types (cervical, uterine, ovarian); Korean source; mean 4.2 conditions per regimen. |
| Data | **5c** | Specifically state the date of the oldest and newest item of text used in the development process (training, fine-tuning, reward modeling) and in the evaluation datasets. | All | All | Yes. HIRA guideline version dated February 1, 2026 (single guideline version). |
| Data | **5d** | Describe any data pre-processing and quality checking, including whether this was similar across text corpora, institutions, and relevant socio-demographic groups. | All | All | Yes. Under the primary condition the original HIRA PDFs were provided to models without preprocessing |
| Data | **5e** | Describe how missing and imbalanced data were handled and provide reasons for omitting any data. | All | All | Yes. Cases were synthetically constructed with a balanced 3-class design (74 per class). |
| **Methods — Analytical Methods** | | | | | |
| Analytical Methods | **6a** | Report the LLM name, version, and last date of training. | All | All | Yes. API model identifiers reported (gemini-3.1-pro-preview, gemini-3-flash-preview, claude-opus-4-6, claude-sonnet-4-6, gpt-5.4-2026-03-05, gpt-5-mini-2025-08-07). |
| Analytical Methods | **6b** | Report details of LLM development process, such as LLM architecture, training, fine-tuning procedures, and alignment strategy and goals. | M, D | All | Not applicable. This study evaluates pre-existing LLMs without fine-tuning or alignment work. |
| Analytical Methods | **6c** | Report details of how text was generated using the LLM, including any prompt engineering (including consistency of outputs), and inference settings (e.g., seed, temperature, max token length, penalties), as relevant. | M, D, E | All | Yes. Standardized prompt template, JSON schema; provider documented default settings used. |
| Analytical Methods | **6d** | Specify the initial and post-processed output of the LLM (e.g., probabilities, classification, unstructured text). | All | All | Yes. Structured JSON containing a 3-class decision (eligible / ineligible / undeterminable) and a single-sentence rationale. |
| Analytical Methods | **6e** | Provide details and rationale for any classification and, if applicable, how the probabilities were determined and thresholds identified. | All | C, OF | Yes. the JSON `decision` field was used directly as the predicted class; no probability thresholds applied. Final per-case prediction determined by majority vote over 3 runs. |
| **Methods — LLM Output** | | | | | |
| LLM Output | **7a** | Include metrics that capture the quality of generative outputs, such as consistency, relevance, and accuracy, compared to gold standards. | All | QA, IR, DG, SS, MT | Yes. Consistency (3 runs) and accuracy. |
| LLM Output | **7b** | Report the outcome metrics' relevance to downstream task at deployment time and, where applicable, correlation of metric to human evaluation of the text for the intended use. | E, H | All | Yes. per-class recall is highlighted as the deployment-critical metric for reimbursement review. |
| LLM Output | **7c** | Clearly define the outcome, how the LLM predictions were calculated (e.g., formula, code, object, API), the date of inference for closed-source LLMs, and evaluation metrics. | E, H | All | Yes. LLM Evaluation reports API-based inference with provider model identifiers (carrying snapshot dates) in March 2026. Statistical Analysis lists metrics (accuracy, precision, recall, F1, 95% CI). |
| LLM Output | **7d** | If outcome assessment requires subjective interpretation, describe the qualifications of the assessors, any instructions provided, relevant information on demographics of the assessors, and inter-assessor agreement. | All | All | Yes. 3 gynecologic oncology experts (2 nurse practitioners, 1 physician) and 1 utilization review nurse; Cohen's κ = 0.93 between experts and review nurse. |
| LLM Output | **7e** | Specify how performance was compared to other LLMs, humans, and other benchmarks or standards. | All | All | Partial Yes. 6 LLMs compared head-to-head with majority voting across 3 runs and 95% CI. Ground truth labels were established by multiple clinical experts and a utilization review nurse (95.0% agreement); a separate human performance baseline for direct LLM-vs-human comparison was not collected. |
| **Methods — Annotation** | | | | | |
| Annotation | **8a** | If annotation was done, report how text was labeled, including providing specific annotation guidelines with examples. | All | All | Yes. Table 1 (definitions and worked example for cervical-cancer topotecan) and Figure 2 (tri-state aggregation rule). |
| Annotation | **8b** | If annotation was done, report how many annotators labeled the dataset(s), including the proportion of data in each dataset that were annotated by more than 1 annotator, and the inter-annotator agreement. | All | All | Yes. All 222 cases reviewed by 3 gynecologic oncology experts and independently by 1 utilization review nurse; initial agreement Cohen's κ = 0.93. |
| Annotation | **8c** | If annotation was done, provide information on the background and experience of the annotators or characteristics of any models involved in labelling. | All | All | Yes. 2 nurse practitioners in gynecologic oncology, 1 attending gynecologic oncology physician, and 1 utilization review nurse experienced in HIRA pre-claim review. |
| **Methods — Prompting** | | | | | |
| Prompting | **9a** | If research involved prompting LLMs, provide details on the processes used during prompt design, curation, and selection. | All | All | Partial Yes. Standardized prompt template designed from the formal definitions of the three outcome classes; held constant across all models and conditions. |
| Prompting | **9b** | If research involved prompting LLMs, report what data were used to develop the prompts. | All | All | Yes. A prompt was derived from the outcome-class definitions in Table 1; no separate prompt-development dataset was used. |
| **Methods — Other** | | | | | |
| Summarization | **10** | Describe any preprocessing of the data before summarization. | All | SS | Not applicable. Not summarization task. |
| Instruction tuning / Alignment | **11** | If instruction tuning/alignment strategies were used, what were the instructions, data, and interface used for evaluation, and what were the characteristics of the populations doing evaluation? | M, D | All | Not applicable. Models were evaluated as deployed by their providers, without additional study-specific instruction tuning or alignment. |
| Compute | **12** | Report compute, or proxies thereof (e.g., time on what and how many machines, cost on what and how many machines, inference time, FLOPs), required to carry out methods. | M, D, E | All | Partial Yes. All inference performed via provider APIs (no local compute); approximate API call volume per model = 222 cases × 3 runs × conditions. Per-case inference cost estimated at approximately USD 0.002–0.10 across the 6 models. |
| Ethical Approval | **13** | Name the institutional research board or ethics committee that approved the study and describe the participant-informed consent or the ethics committee waiver of informed consent. | All | All | Not applicable. The study did not involve human participants, real patient data, or PHI; all scenarios synthetically constructed from publicly available HIRA guidelines; therefore, IRB review was not required. |
| Open Science | **14a** | Give the source of funding and the role of the funders for the present study. | All | All | Yes. No external funding was received for this study. |
| Open Science | **14b** | Declare any conflicts of interest and financial disclosures for all authors. | All | All | Yes. Declared in the Conflicts of Interest section. |
| Open Science | **14c** | Indicate where the study protocol can be accessed or state that a protocol was not prepared. | H | All | Not applicable. Synthetic, no human subjects. |
| Open Science | **14d** | Provide registration information for the study, including register name and registration number, or state that the study was not registered. | H | All | Not applicable. Synthetic, no human subjects. |
| Open Science | **14e** | Provide details of the availability of the study data. | All | All | Yes. Benchmark dataset will be made publicly available on GitHub and Zenodo upon publication. |
| Open Science | **14f** | Provide details of the availability of the code to reproduce the study results. | All | All | Yes. Prompt templates and evaluation scripts will be made publicly available alongside the dataset. |
| Public Involvement | **15** | Provide details of any patient and public involvement during the design, conduct, reporting, interpretation, or dissemination of the study or state no involvement. | H | All | Not applicable. No patients or members of the public were involved in the design, conduct, reporting, interpretation, or dissemination of this study. |
| **Results** | | | | | |
| Participants | **16a** | When using patient/EHR data, describe the flow of text/EHR/patient data through the study, including the number of documents/questions/participants with and without the outcome/label and follow-up time as applicable. | E, H | All | Not applicable. Synthetic cases derived from publicly available reimbursement guidelines; no patient or EHR data used. |
| Participants | **16b** | When using patient/EHR data, report the characteristics overall and, for each data source or setting, and for development/evaluation splits, including the key dates, key characteristics, and sample size. | E, H | All | Not applicable. No patient/EHR data. |
| Participants | **16c** | For LLM evaluation that include clinical outcomes, show a comparison of the distribution of important clinical variables that may be associated with the outcome between development and evaluation data, if available. | E, H | All | Not applicable. No patient/EHR data; benchmark composition characterized in Table 2. |
| Participants | **16d** | When using patient/EHR data, specify the number of participants and outcome events in each analysis (e.g., for LLM development, hyperparameter tuning, LLM evaluation). | E, H | All | Not applicable. Synthetic data. Per-condition sample sizes (n=222 cases per model per condition) reported throughout Results. |
| Performance | **17** | Report LLM performance according to pre-specified metrics (see item 7a) and/or human evaluation (see item 7d). | All | All | Yes. Results, Statistical Analysis, Analysis by Outcome Class, Analysis by Cancer Type, Error Analysis, Sensitivity Analysis; Tables 3–5; Multimedia Appendices 2–6. |
| LLM Updating | **18** | If applicable, report the results from any LLM updating, including the updated LLM and subsequent performance. | All | All | Not applicable. No LLM updating performed. |
| **Discussion** | | | | | |
| Interpretation | **19a** | Give an overall interpretation of the main results, including issues of fairness in the context of the objectives and previous studies. | All | All | Yes. Principal Results and Comparison with Prior Work. |
| Limitations | **19b** | Discuss any limitations of the study and their effects on any biases, statistical uncertainty, and generalizability. | All | All | Yes. Cancer-type and version scope, synthetic structured cases, structural-element errors, default settings without fine-tuning. |
| Usability | **19c** | Describe any known challenges in using data for the specified task and domain context with reference to representation, missingness, harmonization, and bias. | E, H | All | Yes. Incomplete documentation in real-world records explicitly modeled by the undeterminable class. |
| Usability | **19d** | Define the intended use for the implementation under evaluation, including the intended input, end-user, level of autonomy/human oversight. | E, H | All | Yes. Positioned as a supervised decision-support tool for utilization review nurses and clinicians; not as an autonomous adjudicator. |
| Usability | **19e** | If applicable, describe how poor quality or unavailable input data should be assessed and handled when implementing the LLM, i.e., what is the usability of the LLM in the context of current clinical care. | E, H | All | Yes. Information gap-filling pattern shows current LLMs resolve missing information toward eligibility rather than abstaining; deployment requires human review of cases where information is incomplete. |
| Usability | **19f** | If applicable, specify whether users will be required to interact in the handling of the input data or use of the LLM, and what level of expertise is required of users. | E, H | All | Yes. LLM output requires verification by a utilization review nurse or clinician with familiarity in the relevant reimbursement criteria. |
| Future Work | **19g** | Discuss any next steps for future research, with a specific view to applicability and generalizability of the LLM. | All | All | Yes. Expansion to additional cancer types and guideline versions, evaluation on real clinical documentation, agentic and neuro-symbolic approaches. |
